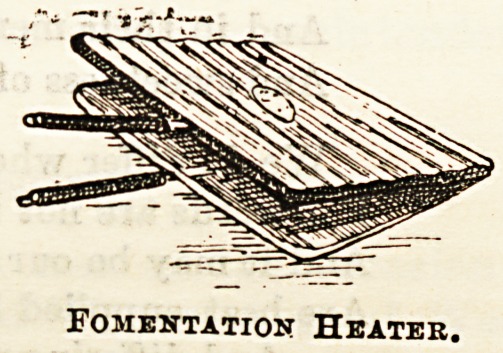# The Hospital Nursing Supplement

**Published:** 1895-11-02

**Authors:** 


### "The Hospital" Nursing Mirror.

Being the Extra Nursing Supplement of "The Hospital" Newspaper.

[Contributions for this Supplement should be addressed to the Editor, The Hospital, 428, Strand, London, W.C., and should have the word "Nursing" plainly written in left-hand top corner of the envelope.]

### News from the Nursing World.

### THE ROYAL NATIONAL PENSION FUND.

The garden party which the Princess of Wales gave to her nurses in July made the end of the London season specially memorable to most of our readers. Her Royal Highness's return from Copenhagen to Marlborough House on Saturday was distinguished by yet another important event connected with this Fund. The Prince of Wales informed the President, "Our Princess," that gifts amounting to £23,500 had been made during her absence, which increased the donation bonus fund to £63,500. The Prince and Princess were delighted by these munificent donations to the Pension Fund, in which they take so warm an interest. Tidings of the safe return of Her Royal Highness, who is said to be in excellent health, were gladly received by her nurses, who realised the vexatious delays caused by the fog during the voyage. The tidings of the betrothal of Princess Maud of Wales to Prince Charles, second son of the Crown Prince of Denmark, have also caused quite a pleasant excitement, and we are asked by numerous nurses to convey their cordial and respectful congratulations to their Royal Highnesses.

### THE PRINCE OF WALES AT NEWMARKET UNION.

A visit was paid to Newmarket Union last week by the Prince of Wales. After inspecting the church which has been erected for the inmates, and also the wards, His Royal Highness wrote in the house visitors' book: "I have visited the Newmarket Union Workhouse with great pleasure and interest, and found the whole establishment in excellent order, the inmates well cared for, and the rooms clean and airy."

### PRINCESS LOUISE AT EDINBURGH.

Princess Louise, president of the Scotch branch of Queen Victoria's Jubilee Institute, visited the Edinburgh Home last week, and presented badges and certificates to the Queen's Nurses, of whom there was a large gathering. They had assembled from all parts of Scotland, and the Marquis of Lorne expressed in a kindly speech the pleasure of Her Royal Highness at meeting so large a party of them.

### OUR NEEDLEWORK COMPETITION.

We want our readers to bear in mind our Christmas needlework competition, and to help us in every way to make it a great success. Each year we have the pleasure of handing on the warm garments made by our friends to patients who spend the festival in hospital wards. Many parcels of well-cut and neatly-made garments reach us, and they furnish much comfort to the patients, who are otherwise badly equipped for exchanging the shelter of a hospital for inclement winter weather. We could always find institutions to welcome any number of useful clothes, and we hope this Christmas will be an exceptionally good competition. Any communication on this subject should be addressed "Needlework," care of Editor of The Hospital, 428, Strand, London. The prizes offered are: One guinea for the best flannel dressing-gown, 10s. for best bed jacket (for man or woman), 10s. for best flannel shirt, 5s. for best flannel petticoat, 5s. for best over-petticoat, 5s. for best knitted socks, 2s. 6d. for second best pair, 2s. 6d. for best warm vest (for man or woman). Other kinds of garments can also be sent to us for distribution, and should be marked "Not for competition."

### PADDINGTON GREEN CHILDREN'S HOSPITAL.

If the plans for Paddington Green Children's Hospital were in some degree cramped by the limitation of the site, in the wards themselves there is no sign of curtailment. They are very lofty and well warmed and ventilated, and the children look so cheerful and happy in their cots that visitors find it hard to think of them as patients. The lavatories and bathrooms are well placed but inconveniently small. Besides the two large wards, one of which is uniquely adorned by large pictures in glazed tile ware illustrative of nursery rhymes, there is a small isolation department for doubtful cases. This is at the top of the hospital, and quite apart from the other wards, and it has an extensive view from all the windows. There is a little room for a nurses' kitchen, &c. The nurses have a pleasant sitting-room, and each has her own tiny bed-room, containing a fire-place and window, and, small as these apartments are, probationer as well as nurse rejoices in "a place to herself."

### A TRAGIC END.

Laudanum, self-administered, is reported as the cause of a young lady's recent death in the neighbourhood of Birmingham. She was the daughter of a Wesleyan minister in Shropshire, and went some six weeks ago as probationer-nurse to the Princess Alice Orphanage, at Erdington. It is said that she suffered frequently from pains in the head, and was in the habit of seeking relief through narcotics. If this statement be true, it seems most extraordinary that the poor girl should have been permitted to take up work for which she was physically unfit. It was also obviously unjust to the orphans to place with them a probationer whose sufferings must have been unwittingly increased by her charges.

### THE HOSPITAL FOR WOMEN AT BIRMINGHAM.

A sale of work under most distinguished patronage is announced to take place at Birmingham on November 8th and 9th, in aid of the building fund of the Hospital for Women at Sparkhill. Various musical entertainments and other attractions will be provided on both days.

### THREE YEARS' TRAINING.

When visiting a very small institution the other day, a correspondent tells us that she was struck by the self-congratulatory remark of one of the staff,

"We have three years' training here," and was puzzled to see what advantages could accrue therefrom. She saw dull wards, untidy quilts, a grubby operating-room, stuff dresses worn by nurses, and lavatories and ward kitchens in undesirable proximity, and says she asked herself, "What is training?" We have so often occasion to answer similar questions that we are inclined to ask our correspondent to study her HOSPITAL, and then she will see that candidates are always advised to enter recognised nurse training schools attached to general hospitals or infirmaries where there are over a hundred beds. Another correspondent kindly informs us that our standard is unnecessarily high, as she can get on to the R.B.N.A. register if she has been simply engaged in hospital or infirmary work for three years, the size of the institution being immaterial. We raise no objection to this correspondent joining any association she likes, but we decline either to lower our own standard or to acknowledge that three years of such undefined work can compare favourably with two consecutive years passed in systematic training at any recognised school.

### RATEPAYERS' VICTORY AT COVENTRY.

THE resolution of the guardians to give the inmates of Coventry Workhouse Infirmary a certificated night nurse has been received with general satisfaction. The ratepayers may congratulate themselves on having by their persistence brought about this much-needed reform. The master reported thirty-six deaths in six months in the infirmary, and an average of ninety occupied beds, and the medical officer has waived his objection, although apparently adhering to his opinion that a night nurse is an unnecessary luxury. Several guardians also owned acquiescence for peace sake in an arrangement for which they failed to see any necessity. Mr. Allchurch took a juster view of such a death roll, and said that the fact that the inmates were such helpless sufferers made him anxious that they should render them in their last moments the best possible assistance and comfort they could. It is unusual to find a medical officer unwilling to exchange pauper "sitters up" for competent nurses.

### NATIONAL UNION OF WOMEN WORKERS.

AN interesting paper on "Rural Nursing" was read by Mrs. Edward Clements at the recent conference at Nottingham. She spoke of the Lincolnshire scheme; Lady Baker praised that favoured in Dorsetshire; and Miss C. J. Wood, Mrs. Dacre Craven, and Mrs. Bedford Fenwick discussed the advantages of fully-trained district nurses.

### THE ALTON NURSE SOCIETY.

THE committee of the Alton Nurse Society have proposed a plan for augmenting their finances, which do not appear to be in a very flourishing condition. In addition to the free nursing of cottagers, with which she has been hitherto employed, the nurse is to extend her services to another class of patients, and the latter are to be asked to pay at the rate of threepence an hour. The local press does not enlighten us as to whether the free patients or the paying ones are to be first considered when their claims become conflicting ones. Surely there must be enough liberal people in the Alton district who, by contributions, could entirely maintain a nurse for their poorer neighbours.

### RUGBY DISTRICT NURSING ASSOCIATION.

A VERY satisfactory report of the Rugby District Nursing Association was laid before the subscribers at the annual meeting. Nurse Reid's services have been greatly appreciated by her patients, and loans of nursing appliances made to the association by several ladies have proved most useful. Mr. J. W. J. Vecquerary has succeeded Dr. Percival as president, and much local interest appears to be taken in the work of the district nurse.

### ST. JOHN AMBULANCE AT LEICESTER.

THE annual meeting of the Leicester branch of the St. John Ambulance Association was well attended, and the Mayor, who presided on the occasion, described at some length the work which had been carried out in the town and neighbourhood. The Right Hon. Lord Knutsford spoke of Leicester as one of the best centres of the association, and the classes held in the district appear to have been of much practical value to both men and women.

### NUNS AS NURSES.

IN reporting on the condition of the lunatics at Longford Workhouse the other day, the Inspector commended their condition, duly crediting the nuns with their careful supervision of the pauper attendants. Somewhat illogically, the local Press seeks to show that because the good nuns see that the imbeciles are well tended, therefore their nursing qualifications ought never to be criticised. We do not think that any doubt has been raised as to the invariable kindness exhibited by nuns towards all with whom they come into contact, but the skilled nursing of the sick requires a full course of systematic training, and cannot properly be undertaken in institutions by any one who has not been duly instructed in a nurse-training school.

### NURSES FOR JOHANNESBURG.

THE religious Sisters at the Johannesburg Hospital are to be replaced by thirty fully-trained Englishwomen. The salaries are given, with other particulars, in our advertisement columns this week, and we imagine that there will be many applicants for the vacant posts in South Africa.

### SHORT ITEMS.

THE Alyth and Meigle Nursing Association Committee have voted in favour of the employment of a Queen's nurse.—A Salvation Army "nurse" died in the Mildmay Memorial Hospital from the effect of extensive burns, her dress having caught fire from a gas stove in a patient's room.—A bazaar for the Barry, Cadoxton, and District Nursing Association and the Accident Hospital will take place on December 9th, 10th, and 11th. Contributions are invited.—The Atherstone Nursing Association have received £24 as a result of the first year's proceeds of the association's football challenge cup.—On leaving the Aberdeen West Poorhouse Hospital to be married, Nurse J. Cornfoot was presented by the officials with a handsome timepiece.—Nurse Rae has made 2,901 visits to 102 cases in the course of the year, and the Jedburgh Nursing Association is popular and prosperous.—The Matrons' Council discussed the subject of uniform training at the meeting last week.

### Elementary Physiology for Nurses.

By C. F. Marshall, late Surgical Registrar Hospital for Sick Children, Great Ormond Street.

### XIII.—THE NERVOUS SYSTEM—(*continued*).

### Functions of the Spinal Cord.

(1) Section of the spinal cord causes complete paralysis, both of motion and sensation, in the parts below the point of section. (2) Irritation of the distal end causes movement of all muscles supplied by nerves arising from the cord below the point of section. Irritation of the proximal, or upper end, causes pain. So far the spinal cord appears as a large nerve of mixed motor and sensory function, and if we pursue our experiments and make sections of the spinal cord higher and higher we shall obtain similar results. It therefore follows that the brain, and not the spinal cord, must be the centre whence all motor impulses originate, and where all sensory impressions are felt. For if the nervous connection between the brain and a part of the body is cut anywhere, voluntary power of movement and sensation are lost at once in this part. Thus, when you bend your finger the motor impulse starts somewhere in the brain, travels down the spinal cord and along the nerves going to the muscles which move the finger, and causes them to contract. Again, when you pinch your finger, the irritation in the finger is propa-

Fig 8.—L and R, left and right sides of brain; M, motor nerves; S sensory nerves.

gated along sensory nerves to the spinal cord, and up to the brain, and it is not till the brain is reached that pain is felt. If the nerve connection is severed anywhere along the track no pain is felt; while if the track is disturbed or irritated at any point pain will be felt in the finger. We may illustrate this by comparison with telegraph wires and a telegraph office. Supposing there is a central office in London with wires radiating in all directions to the surrounding towns, separate wires going to each town. The telegraph operator in London would from long practice get into the habit of associating a message received along a particular wire with a special locality, and would refer a message received by that wire to that locality. Now, if the wire supplying this locality was cut, and a message sent from an intervening point, the telegraph operator would still think the message came from the town where the wire was cut. So it is with nerves, as we have seen in cases of pain after amputation, caused by irritation during the process of healing, and referred to the parts formerly supplied by those nerves.

So far we have considered the spinal cord as a mere conductor of impulses. We have now to define more correctly the path along which the impulses travel. If the spinal cord is cut half-way across, say on the right side, there results motor paralysis on that side, and sensory paralysis on the left side. Therefore the sensory nerves of the right side must run up the left side of the spinal cord. The fact is that all the nerves cross over to the opposite side of the body after passing out of the brain and spinal cord. The sensory nerves cross over as they enter the spinal cord; the motor nerves cross over in the medulla oblongata, or lower part of the brain. Thus the right half of the body is connected with the left half of the brain, and *vice versa*. This is shown in the accompanying diagram.

### The Functions of the Brain.

We have already seen that the brain is the real centre for both voluntary motions and sensations. That we really feel in our brains is evident from the effect of a blow on the head or from a dose of chloroform, under which circumstances the brain is sent to sleep for a time, while the nerves and muscles are not affected. The brain is also the centre for all the higher emotional and intellectual activities, it is the seat of the mind, also of the special sensations of sight, hearing, &c., and it is in the brain that the nerve centres governing the respiratory movements are situated.

*The Cerebral Hemispheres.*—If the cerebral hemispheres are removed from a frog, the effect is that the frog shows no voluntary movement. It will respond to the stimulus of irritation, but does nothing of its own accord. It breathes naturally, and will sit on a table in a natural position. When thrown into water it swims vigorously until a landing place is found. When placed on its back it turns over at once. It croaks when its flanks are touched. In fact its movements are the same as in an uninjured frog, except that they require an external stimulus to call them forth. If the whole brain is removed from a frog it does not breathe, but lies flat on a table in an unnatural position. When thrown into water it sinks like lead, and when placed on its back remains there without an effort to replace itself.

The functions of the central hemispheres in man are known directly only from the effects of wounds or disease; indirectly from comparison with animals. The parts of the surface specially associated with the movements of the leg, arm, face, and trunk, and other parts associated with sight and hearing have been localised by the effects of disease and by comparison with lower animals. The anterior part, or the frontal lobes of the brain, are probably the seat of the higher intellectual powers. In the medulla oblongata, the lowest part of the brain where it joins the spinal cord, are a large number of nervous centres of great importance; viz., the centre governing respiration the vaso-motor centre governing the arterial system, and other centres governing the actions of coughing, vomiting, swallowing, &c.

### Minor Appointments.

Union Infirmary, Northampton.—Miss Fanny T. Daniel has been made Head Nurse at this infirmary. She was trained at Berrywood Asylum, and by Sister Katherine at Plaistow, where she obtained a certificate for monthly nursing. On leaving Berrywood Miss Daniel was presented with a handsomely-fitted midwifery bag by her fellow nurses and attendants, also a desk and album and various other gifts from the officers, accompanied by sincere wishes for Miss Daniel's prosperity. We congratulate her on her promotion.

### Wants and Workers.

Does any reader of The Hospital know of a person willing to adopt one of twin girls, a fortnight old, pretty, and healthy, All particulars from Miss Lawrie, Garrison Nurse, 9, St. Martin's Terrace, Dover.

Sister Katherine, Howards Road, Plaistow, E., will be very glad of flannel jackets, night-gowns, and dressing-gowns (new or old) for the sick poor of the district. The cold weather brings much suffering, and Sister Katherine's cupboards are almost empty.

### Twenty-three Thousand Pounds for the Pension Fund.

THIRD CONTRIBUTION OF £23,500.

Another £23,500 for the Royal National Pension Fund for Nurses! What does this mean? Well, in the first place, that the members of the Fund will get, in the most desirable interpretation of that dubious phrase, more than they bargained for; second, that there are those who have the will, the power, and the energy to help them; thirdly, that there are rich men who are willing to give—might not one even say anxious to give?—to any object which they are sure is useful and good.

When the Pension Fund was first established one of the favourite arguments used against it by a party who perhaps meant, as they professed, good to the nurses, but certainly meant harm to the nurses' fund, was that no nurse could put by out of her earnings enough to secure for her, at the rates allowed by the actuary of the fund, an annuity sufficient to keep her in comfort after her working days were past. There was a certain measure of truth in the statement, but the founders of the Pension Fund said, "Take out a policy for as much as *you* can afford, and trust to us to make it as much larger as *we* can afford." The proposal was scoffed at by the persons we speak of, although it is carried out every day by many of our most reliable insurance companies, and the bonus additions to a policy amount often to a substantial sum. But in no company has it been carried out on such a magnificent scale as in the Royal National Pension Fund for Nurses. For not only has that Fund the advantage of having the contributions of its members managed by some of the soundest and shrewdest business men in London, but the gifts of friends who are interested in nurses are added to their own savings.

Everyone knows of the magnificent donations which started the Fund, but the interest of that, large though it was, would not have availed to have added as largely as was desirable to the policies taken out by a constantly increasing number of nurses. Therefore, realising this, the founder saw the importance of raising the Donation Bonus Fund to such a sum that all who joined might be equally benefited. It has ever been held by him that there are plenty of people willing to give, if they knew any object to give to which was not tainted with imposture, nor tended to pauperise. He has proved again and again that the rich men of England are generous. Witness the sum just handed over to the Lord Mayor for a charitable object. That, however, represents only a part of the sum raised since last June. Altogether £45,500 has been collected, of which £23,500 goes to the Donation Bonus Fund of the Royal National Pension Fund for Nurses. All honour to those whose large-hearted generosity has relieved nurses from the fear that a life of unselfish labour might end in poor and dependent old age! Now they may reasonably hope that their declining days—the rest that comes after toil and precedes sleep—will be "serene and bright, and lovely as a Lapland night." We subjoin a list of the contributors to the £23,500—the third sum of that amount collected since the Fund started, in addition to the £30,000 given to set it on its feet; and it will be seen that while new names appear on the list, showing that new friends are being gained for the Fund, those of the first donors and their families reappear, showing that those who gave in the beginning are satisfied with the conduct and results of the Fund, and that the limit of their interest in nurses and kindness to them has not yet been reached. Special thanks are due to Mr. Walter H. Burns (the chairman of the Fund), who himself has collected the munificent sum of £5,000 on this occasion. Mr. W. H. Burns is the representative of the Morgan family, whose name can never be forgotten by any nurse in the British Empire.

Donors to the Donation Bonus Fund.

| | |
|---|---|
| Mr. J. B. Robinson | £5,000 |
| Messrs. Wernher, Beit, and Co. | 5,000 |
| Mr. Walter H. Burns (a further) | 2,000 |
| Lord Iveagh | 2,000 |
| Mr. J. Pierpoint Morgan (a further) | 2,000 |
| Messrs. N. M. Rothschild and Sons (a further) | 1,600 |
| Mr. D. Marks | 1,105 |
| Messrs. Marks, Bulteel and Co. | 1,000 |
| Mr. S. Neumann | 1,000 |
| Mr. E. A. Hambro (a further) | 500 |
| Messrs. L. Hirsch and Co. | 500 |
| Messrs. Ansell, Mankiewicz and Tallerman | 300 |
| Mr. E. Cassel | 250 |
| Mr. Robert Gordon (a further) | 200 |
| Mr. G. Bulteel | 105 |
| The Hon. Egremont Mills | 105 |
| Messrs. De La Bere, Bellairs, and Pelham | 100 |
| Mr. Carl Hanau | 100 |
| Messrs. Hyam Brothers | 100 |
| Mr. D. J. MacRae | 100 |
| Mr. C. Morrison (a further) | 100 |
| Mr. Harry Pearson | 100 |
| Mr. H. L. Raphael | 100 |
| Mr. E. Rawlings (a further) | 100 |
| The Duke of Northumberland | 25 |
| Mr. Jeffery Whitehead | 10 |
| | £23,500 |

How to Thank the Merchant Princes.

The grateful thanks of all nurses are due to each and all the donors for their munificent gifts. It is difficult to suggest how such acknowledgments can be expressed, but if there be a general feeling in favour of such a step we shall be glad to prepare an address to the donors. It can then lie for signature at our offices, 428, Strand, for a fortnight, so that nurses in London may call and inscribe their names, whilst those unable to do so may have their names entered for them if they will send us a request to that effect. We will make a definite statement on this point next week, and meanwhile anyone may express their views on the point by letter addressed to the editor.

### Where to Go.

Royal British Nurses' Association.—The first sessional lecture of the season will be given at 17, Old Cavendish Street, on Friday, November 22nd, at eight p.m., by Mr. Louis H. Parkes, M D., D.P.H., medical officer of health, Chelsea, on "The Importance of Breathing Fresh Air." Admission to members, free; to the public, 1s.

Chrysanthemum Show.—The National Chrysanthemum Society will hold their great show on November 5th, 6th, and 7th at the Royal Aquarium.

Sale of Work and Entertainments on November 8th and 9th in aid of the building fund of the Birmingham and Midland Hospital for Women at Sparkhill. Sale will be opened by the Countess of Dudley, president of the hospital.

### Appointments.

[It is requested that successful candidates will send a copy of their applications and testimonials, with date of election, to The Editor, The Lodge, Porchester Square, W.]

Lewisham Workhouse Infirmary.—Miss Emmie Lofts has been appointed Matron of the Lewisham Infirmary. Miss Lofts was trained at St. Bartholomew's Hospital, and from thence went to the Chelsea Infirmary as night superintendent. She subsequently became assistant matron, and takes many good wishes with her to a post for which her experience at Chelsea has been an excellent preparation.

### Novelties for Nurses.

An excellent exhibition of nursing appliances was opened at the Trained Nurses' Club, 12, Buckingham Street, Strand, on Friday last, and remains on view for a fortnight. The collection there gathered together is most comprehensive, and the number of really new inventions and practical contrivances for the sick room and hospital ward quite surprisingly large. Miss Brierley, the Editor of *Nursing Notes*, and her energetic co-workers must be cordially congratulated on the success which has attended theire ffurts, for the result is a most interesting exhibition, and no pains have been spared to make it as complete as possible; nothing would appear to have been admitted which has not some practical recommendation. Nurses will find numberless hints to help them in their daily work, and all who can should avail themselves of so good an opportunity to pick up crumbs of useful knowledge.

Among so much that is interesting the difficulty is to pick out the exhibits most worthy of special mention for the benefit of those who may be unable to see for themselves. Messrs. Down Bros. show a number of new appliances of various kinds. The "Priestly Smith" candle lamp, illustrated above, is a useful invention, and for district work especially will be a boon to nurse or doctor. This is made after the fashion of a carriage lamp, but small enough to go

Ward Lamp at Poplar Hospital.

Pocket Candle Lamp.

Fomentation Heater.

in pocket or hand-bag. It may be telescopic or not, is convenient to hold, and quite steady when placed on the table. Amongst Messrs. Down's things we noticed the useful little "Sister Louise" ice cup, of which we gave a description a few weeks ago in these columns; also the new irrigating dressing tray with indiarubber tube, recently commented upon. Messrs. Down show glass catheters, which are now looked upon with great favour by many doctors and nurses, glass being aseptically preferable to any other substance; and they also show an excellent sterilizer, a compact little apparatus with tiny spout which can be heated over a spirit lamp, the spout placed in the end of the catheter, and the steam passing through and escaping at the eye. Dr. Hale White's tongue depressor, fitted with a glass shield for use in cases of diphtheria, is an invaluable invention. Diphtheria has been contracted many a time through the coughing up of expectoration during throat examination, and by this simple shield, fixed to a Türck's spatula, the danger is entirely obviated. The glass can be detached for disinfecting, and the spatula is entirely made of metal, and so allows of boiling. An excellent spittoon was another noticeable article on Messrs. Down's stall, consisting of a metal frame and cover, fitted with glass cup, delightfully cleanable.

A very good safety lamp for ward use comes from the Poplar Hospital for Accidents, where it is in favour. It is nickel-plated; the receiver is capacious, and with the aid of the reflector a very strong light is obtained. It is of a particularly firm and steady make, as will be seen by the sketch given above. The third illustration represents quite a novelty; it is to be found on Messrs. Bailey and Sons' stall, and is universally approved of by those nurses who have made its acquaintance. It is "Hackforth's Patent Heater for Fomentations," of thin galvanized iron or block tin, a splendid medium for saving the nurse's hands in the preparation of poultices and fomentations, and for ensuring their application at a proper degree of heat. The damped flannel for a fomentation, folded to the required size, and placed inside the heater, can be held over fire or spirit lamp, and be ready for use in a few minutes. Messrs. Bailey's electric call bells also excited our admiration. For private nursing they must be the greatest convenience. In many cases where a patient may need to call the nurse during the night from an adjoining room there is often much difficulty experienced in arranging a method of summons. This compact little battery, with a bell sharp enough to arouse even a sound sleeper, can be placed by the nurse's bed, and the end of the wire with its knob and pressure button within the invalid's reach. Yards of wire are attached, so that it would be possible to arrange a communication for almost any distance, under a door or through a keyhole. The moderate price (17s. 6d.) brings this useful little apparatus within the compass of most people.

A steam-kettle for disinfecting purposes, used in the lying-in ward of St. George's Workhouse, lent by Mrs. Dillon, has many practical recommendations, and has proved valuable in practice. A wonderful number of ingenious contrivances come from the Mildmay Mission Hospital, but these and others must be left till next week for description. We must say, however, one word of admiration for the complete "ward," sent from the Evelina Hospital, with its beds and their occupants, all in miniature, the doctor with a tiny stethoscope round his neck, sister and nurse, the doctor's table with its jug and basin, towels and soap dish, all complete, down to the smallest detail.

### Everybody's Opinion.

### NURSES' UNIFORMS.

"A Nightingale Nurse" writes: May a true nurse express through your columns her great surprise at the remarks recently made by a superintendent in the Hospital? Surely if this superintendent has been a nurse herself she does not wish to see the Sairey Gamp days again. If our large institutions would supply a special uniform with a badge always to be worn, these could not be very well copied, and we should be able to distinguish the real nurse from the unreal. The true nurse has no wish to be deprived of her uniform, as she does not care for the fashions of jaunty little shoes, &c. It seems a pity that hotel proprietors consent to take in nurses (as they always have previous knowledge of their arrival), if they cannot permit them to be seen at their tables, &c. I have nursed several times in hotels, and my uniform has not been objected to. Possibly this hotel manager made a mistake in some way, and thought within himself he had to deal with one who "copied our uniform." [Our correspondent appears unaware that many institutions do supply uniform, and some of them have also a badge, but this has not availed to prevent unworthy imitations.—Ed. T. H.]

### HOURS AND WORK OF ASYLUM ATTENDANTS.

"A Lover of Justice" writes: Having had experience as a nurse in a private asylum I shall be glad if the Editor will kindly let me give my opinion of the hours and work at the establishment where I was for some little time. Each patient had a nurse to herself, with the exception of four ladies who required very little attention, and they went out in the grounds with a nurse who had not a troublesome patient. The nurses had to see that their patients are bathed, dressed and downstairs in time for breakfast at

9 a.m., certainly not a very early hour. On each floor there was plenty of hot water, and it surely was very little to ask of a nurse to just carry the water into the room. After breakfast the nurses relieved one another and made their patient's and their own beds, dusted and tidied their bedrooms. The sweeping, cleaning of floors, windows, and emptying of water was all done by housemaids. Beyond keeping their patient's clothes in order, that was all the actual work the nurses were expected to do. The nurses whose patients go to the dining-room, took it in turns to be in the room at meal times, in case they might be required. All the lights (electric lights) were supposed to be out by 10.30 or 11 p.m. (I forget which). During the day the nurses had plenty of opportunities of talking to each other; and the patients who were well enough, and did not prefer a private sitting room, had a large cheerful drawing-room, containing a piano, and several games, illustrated papers, magazines, &c. I must say that we nurses had nothing to complain of, though we might have had a more comfortable place for our meals. As I am writing about a time more than two years ago (the doctors of the asylum had only recently removed there), I dare say that is better now. The off-duty time was good, and the doctors and lady superintendent were very kind, and never found fault without occasion. I am sorry that "A Nurse in a Private Asylum," has not had a happier experience, but I do not think it right that girls of a better class (whom I should strongly advise to take up the work) should be prejudiced in such a manner. I hope that others, who have had the good luck to meet with as much kindness as I have, in a private asylum, will also give their experience. I cannot end without saying that a nurse-attendant I have at present with me, and who is also employed at a private asylum a short distance from London, fully endorses all I have written.

### CONVALESCENT HOME FOR WOMEN AND CHILDREN, NEW BRIGHTON.

Miss Forster writes: We are indebted to you for the kind notice of our home in last week's Hospital, and I write to thank you very much, and also to tell you that we have a grand piano in the Recreation Hall, which was presented by Mr. David Grainger, of Liverpool, when the new wing was opened. What I really want now is a very sweet-toned piano for the lady patients' drawing-room. The old one is worn out, and the touch is so heavy it is unsuitable for anyone who is not blessed with very strong fingers.

### THE ABUSE OF UNIFORM.

"A Trained Nurse" writes: I am glad to see some correspondence on the subject of uniform in the columns of your widely-read paper. What do readers of The Hospital think of an advertisement which appeared in a leading daily paper of October 14th: "Woman (young) required for several weeks, commencing 21st inst., to attend counter in chemists' shops, for pushing the sale of a proprietary article; must wear nurse's uniform"?

[Another correspondent sends in the same advertisement, with a letter which we give below.—Ed. T. H.]

"A Lady Superintendent" writes: "I have been advised to send you enclosed advertisement, which was recently cut out of the *Daily Telegraph*, and trust that you will insert it in The Hospital, with a few pointed remarks. We wonder what will "nurses' costume" be used for next? It is a subject on which I feel most strongly, and I heartily wish some means could be adopted to prevent the abuse of our hitherto "respected and respectable dress." Nurses should in a body refuse to wear uniform unless in some way protected from bad imitations. I am a registered member of the R.B.N.A., and also on the General Council.

### For Reading to the Sick.

### THE COMMUNION OF SAINTS.

All Saints' Day, 1895.

**Motto.**

"We lose much of the joy of our heavenly expectation if we do not keep in mind the Communion of Saints."

—*R. M. Benson, "Spiritual Readings."*

**Verses.**

All Saints!—the unknown good that rest,
In God's still memory folded deep:
The bravely dumb that did their deed,
And scorned to blot it with a name,
Men of the plain heroic breed,
That loved heaven's silence more than fame.

Such lived not in the past alone,
But tread to-day the unheeding street,
And stairs to sin and famine known
Sing with the welcome of their feet;
The den they enter grows a shrine,
The grimy sash an oriel burns—
Their cup of water warms like wine,
Their speech is filled with heavenly urns.

About their brows to me appears
An aureole traced in tenderest light,
The rainbow—gleam of smile through tears
In dying eyes, by them made bright—
Of souls that shivered on the edge
Of that chill Ford repassed no more,
And in their mercy felt the pledge
And sweetness of the farther shore. —*Lowell.*

We do differ when we most agree,
For words are not the same to you and me.
And it may be our several spiritual needs
Are best supplied by seeming different creeds—
And differing we agree in one
Inseparable Communion,
If the true Life be in our hearts—the Faith,
Which not to want is Death;
To want is penitence; to desire
Is purgatorial fire;
To hope is Paradise; and to believe,
Is all of Heaven that Earth can e'er receive!
—*Hartley Coleridge.*

**Reading.**

"What a thick veil it is which hangs between us and the unseen world! How we long to pierce through for a moment, and see what the spirits of the saints at rest are doing! It is almost dreadful to know so little about them. At least those who have seen loved ones pass behind that veil, long—they cannot help longing—to know something more. One day with us, sharers in our hopes, our joys, our sorrows, our prayers; the next gone, and all so dim and mysterious and hard to understand! Well, God has willed it to be so. If it were good for us to know more, we should know. There are some plain reasons, which any of us can see, why it might be very bad for us. God wills that the affections and desires and utterances of the soul, which go forth into the world unseen, should centre in Him. I do not mean that He would bid us forget our earthly love, or put away our hope to meet again. But if we knew more, might not this knowledge become the absorbing passion of our souls, and the love and worship of God be dimmed or hidden by it?"—*Bishop W. Walsham How.*

"These are they who have come out of deep tribulation and have washed their robes and made them white in the Blood of the Lamb."—*Revelation* vii. 24—26.

### Royal British Nurses' Association.

### IMPORTANT STATEMENT BY DR. BEZLEY THORNE.

We have received from a reliable source the following report of the statement made by Dr. Bezley Thorne on retiring from the office of honorary medical secretary of the association :—

I beg to inform the General Council that I, this day, lay down the office of medical honorary secretary of the Royal British Nurses' Association. In so doing, it will not be necessary for me to make any statement on the progress and policy of the corporation. I should, however, fail in my duty if, vacating office at this juncture, I made no reference to the sufficiently notorious fact that the course of events with regard to the internal affairs of the corporation has been neither smooth nor unchequered, and offered no explanation of events which have been a cause of perplexity to a large said at once that nothing has arisen in the course of either proportion of the members of the association.

"A Small Unofficial Organization."

It may be deliberation or the transaction of business which might not have been satisfactorily dealt with had the ethical rules which are generally expected to govern the conduct of both social and official intercourse, and respect for the ordinances which have been laid on the corporation for the governance, been duly observed by all concerned. That such has not been the case the members know, and the public have not been slow to learn. What is not equally well understood is that the discord which has been so ostentatiously displayed has been mainly due to the existence within the association of a small and unofficial organisation which aims at forcing its own views and aims, at all costs, and by almost any means, on the governing bodies, and of exercising a dominating influence in the direction of their policy and actions.

"Objects and Methods of the Ruling Minds."

As is the case with similar agencies, the objects and methods of the few ruling minds are mainly kept in the background while adherents and allies, less well informed, are encouraged to come forward on public occasions and otherwise employed to carry out a policy the general tendency of which they do not understand. With regard to the methods employed, I have already stated, in addressing the adjourned meeting of the General Council in May last, that, as directed against non-compliant officials, they may be summarised as obstruction, detraction, and misrepresentation at times of the most flagrant and personal kind; and I stated, as an instance, that the resignation of my former colleague, Miss Gordon, was due to the fact that her health succumbed to the strain induced by the practice of such devices.

An "Occult Organization: Its Chief Aim."

My own chief offence, in the eyes of some, is that I remained at my post, and up to the present hour have stood athwart the path of the occult organisation. It now remains to inquire what is the chief aim of that body. It is, by one means or another, to reduce to impotence in the counsels of the association the medical members and those matrons and nurses who, consistently with the avowed purposes of the association, and with its authorised constitution, persist in regarding the nursing vocation as the subordinate ally of the medical profession. In the determined pursuit of their object, the leaders of the movement have been prepared to sweep out of their course all who have stood in the way, not excepting officers of the corporation from the lowest to the very highest. I am quite aware that it has been strenuously denied that such has been the case, and it probably will be again denied, but if the necessity for so doing were to arise, it would not be difficult to adduce convincing testimony to show that such denial is worthless. Speaking as an original member of the association, and as an executive officer of nearly four years' standing, I venture to assert, for the information of all whom it may concern, that the medical profession will not remain silent or passive spectators of any efforts which may be made to carry such a policy into execution. Be that as it may, it will be well for the members of the General Council, and the members of the association at large, seriously to consider the fact that it has been reserved for men and women who stand pre-eminent in their respective callings, who have for years been engaged in the transaction of public business to the satisfaction of their compeers and to the advantage of their constituents and dependents, some of whom have been considered worthy to receive distinction at the hands of their Sovereign—it has been reserved for such to have their motives and conduct impugned, and to be held up to public defamation, in return for having held out a helping hand of good fellowship to trained nurses in their first effort to organise themselves into a regularly constituted body. Nor should it be overlooked that one result of the tactics to which I have alluded has been to bring this Royal corporation, within the present year, to the very verge of a humiliating disintegration.

### DR. BEZLEY THORNE'S RESIGNATION.

Eugenie Alice Warren writes: As a member of the council of the R.B.N.A., but unable to be present at the meeting on October 18th, I wish publicly to record the dismay with which I and many other members of the association heard of the resignation of our esteemed—honorary—secretary, Dr. Bezley Thorne. His unfailing courtesy, earnest devotion to duty, and unflagging energy in the great nursing cause have won him the esteem and gratitude of all loyal nurses, and his loss will be a blow to the association under which it will reel for some time to come. As I have not had an opportunity of expressing these sentiments at the meeting, I trust in your courtesy you will accord me the space in your valuable paper to do so now.

Miss Josephine L. de Pledge writes: Will you afford me a brief space to give expression to the heartfelt regret with which I read in your columns last week of the resignation of Dr. Bezley Thorne as medical honorary secretary of the Royal British Nurses' Association? I am not serving on the council this year, and so missed an opportunity when it last met of expressing that gratitude to Dr. Thorne which is only his due, after the unceasing energy and zeal he has shown in the affairs of the corporation. I believe I am but expressing the views of many other members when I add that his loss as an officer of the association is one which is very deeply to be deplored.

### "The Hospital" Convalescent Fund.

The hon. secretaries have the pleasure of acknowledging 5s. from an anonymous policyholder and 2s. 6d. from Nurse A. O. Dickinson for The Hospital Convalescent Fund.

### Notes and Queries.

The contents of the Editor's Letter-box have now reached such unwieldy proportions that it has become necessary to establish a hard and fast rule regarding Answers to Correspondents. In future, all questions requiring replies will continue to be answered in this column without any fee. If an answer is required by letter, a fee of half-a-crown must be enclosed with the note containing the enquiry. We are always pleased to help our numerous correspondents to the fullest extent, and we can trust them to sympathise in the overwhelming amount of writing which makes the new rules a necessity. Every communication must be accompanied by the writer's name and address, otherwise it will receive no attention.

### Queries.

(18) *Lady Roberts' Nurses.*—Kindly tell me how I can get information about these nurses.—*A. N. S.*

(19) *Massage.*—Where can I get practice after having recently learnt massage?—*F. R.*

(20) *Medical Women.*—Can you tell me particulars as to age and preliminary study needed in women doctors?—*A Would-be Lady Doctor.*

(21) *Midwifery.*—Where can I get training as midwife and monthly nurse?—*Anxious.*

(22) *Monthly.*—Is it necessary for a midwife to have general hospital training?—*B.*

### Answers.

(18) *Lady Roberts' Nurses* (*A.N.S.*)—"The Hospital Nursing Supplement" p. cxxxvi., July 1st, 1893.

(19) *Massage* (*F. R.*)—You should write, enclosing stamped envelope for reply, to Hon. Secretary, Society Trained Masseuses, 12, Buckingham Street, Strand.

(20) *Medical Women* (*A Would-be Lady Doctor*).—You had better write to the secretary of the School of Medicine for Women, Handel Street, London, W.C.

(21) *Midwifery* (*Anxious*).—Get "How to Become a Nurse" from Scientific Press.

(22) *Monthly* (*B*).—Without general hospital training a midwife is not a nurse. The after prospects of earning a livelihood are largely increased by possession of a hospital as well as a midwifery certificate.

## Figures and Tables

**Fig 8. f1:**
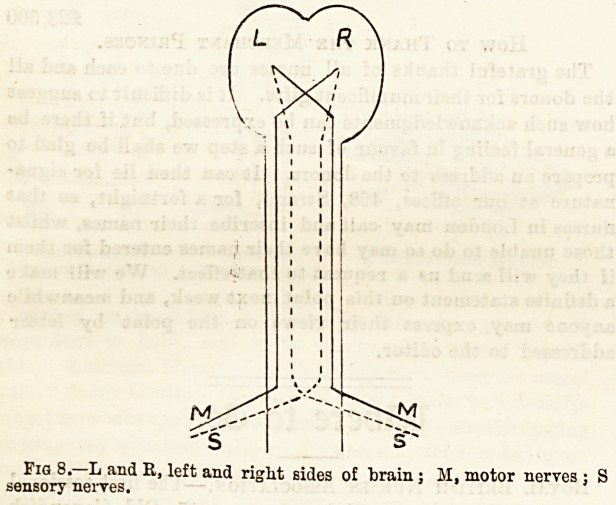


**Figure f2:**
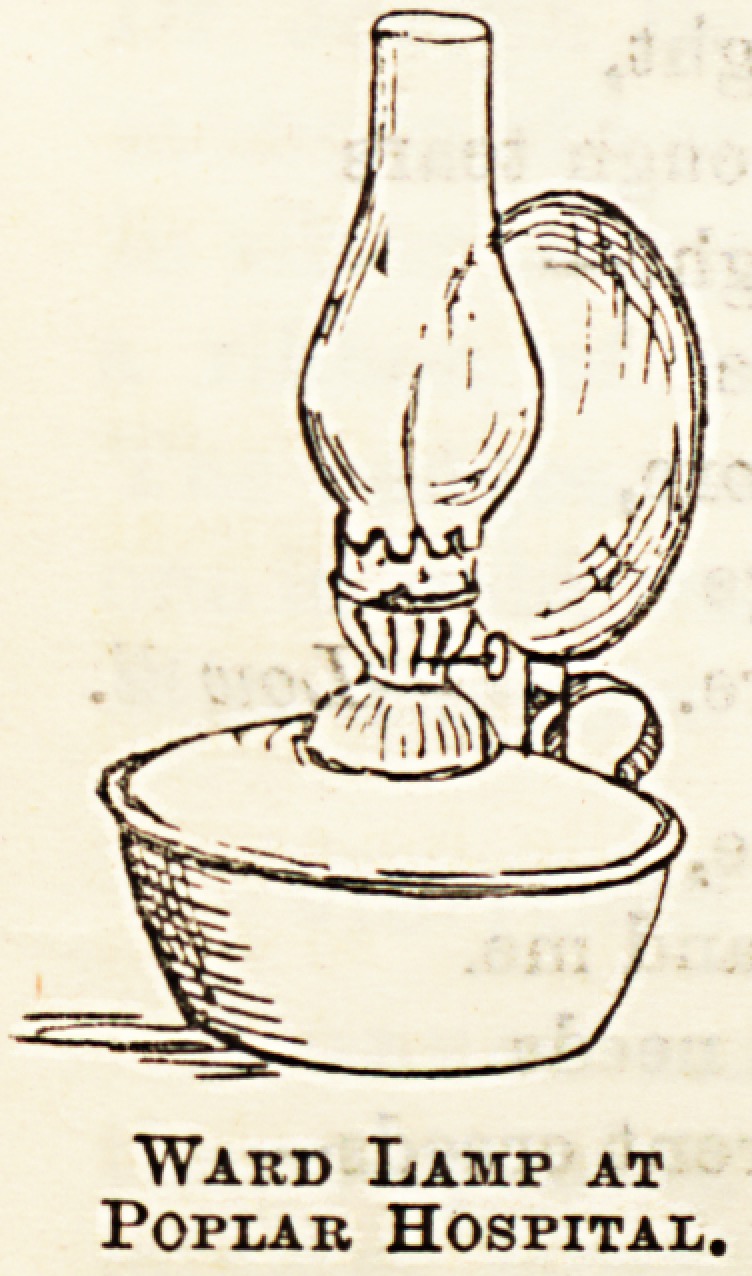


**Figure f3:**
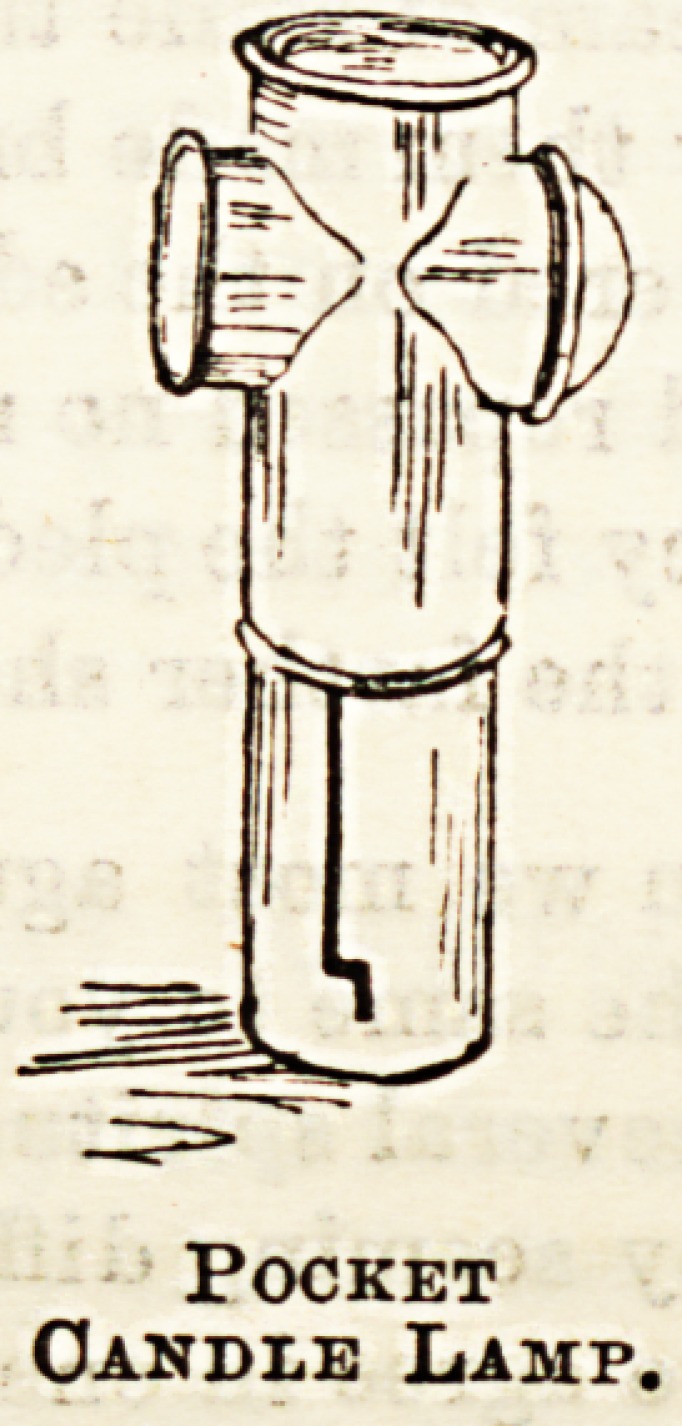


**Figure f4:**